# Supplementary material for: Integrated genomic analysis reveals aberrations in WNT signaling in germ cell tumors of childhood and adolescence
Source: Nat Commun. 2023 May 6;14:2636. doi: 10.1038/s41467-023-38378-9 (PMC10164134; doi:10.1038/s41467-023-38378-9)
Supplement: Supplementary file 1 — Supplementary Information [file 41467_2023_38378_MOESM1_ESM.pdf]

Supplementary Data for Xu et al., "Integrated genomic analysis reveals aberrations in WNT signaling in germ cell tumors of childhood and adolescence"

Contents: **page**

**Supplementary Figures**	**2**
**Supplementary Tables**	**11**
**Supplementary Data Legends**	**22**

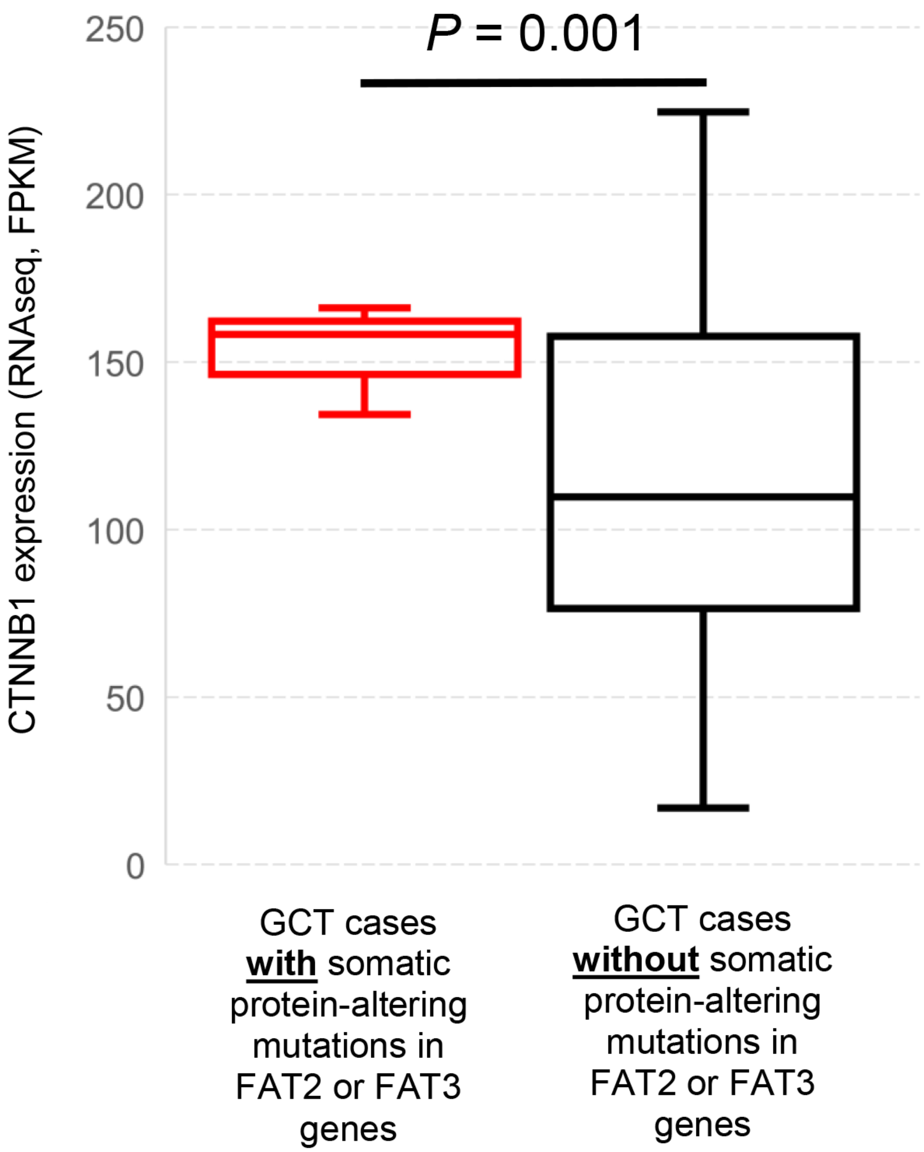

Supplementary Figure 1. *CTNNB1* expression in tumors with and without somatic protein-altering mutations in *FAT2* or *FAT3* genes. n=32 biologically independent tumors. The center line denotes the median value, the box contains the 25th to 75th percentiles and the whiskers mark the 5th and 95th percentiles. Two-sided Student's t-test, p=0.001.

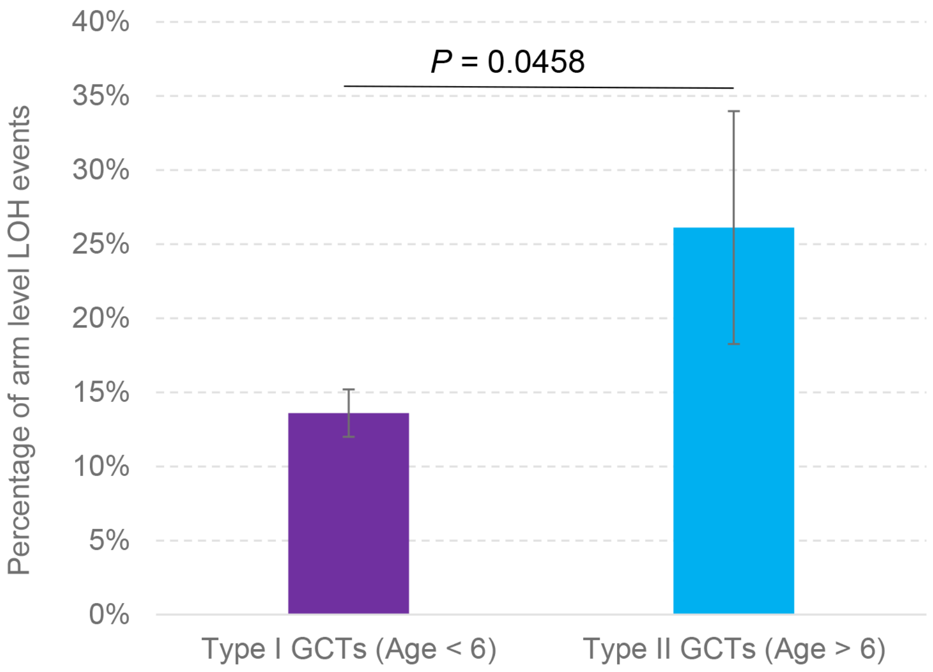

Supplementary Figure 2. Percentage of arm-level loss-of-heterozygosity (LOH) events in type I and type II GCTs, respectively under and above the age of 6 years. n = 148 biologically independent tumors. Data are presented as mean values +/- SEM. Two-sided Student's t-test, p=0.0458.

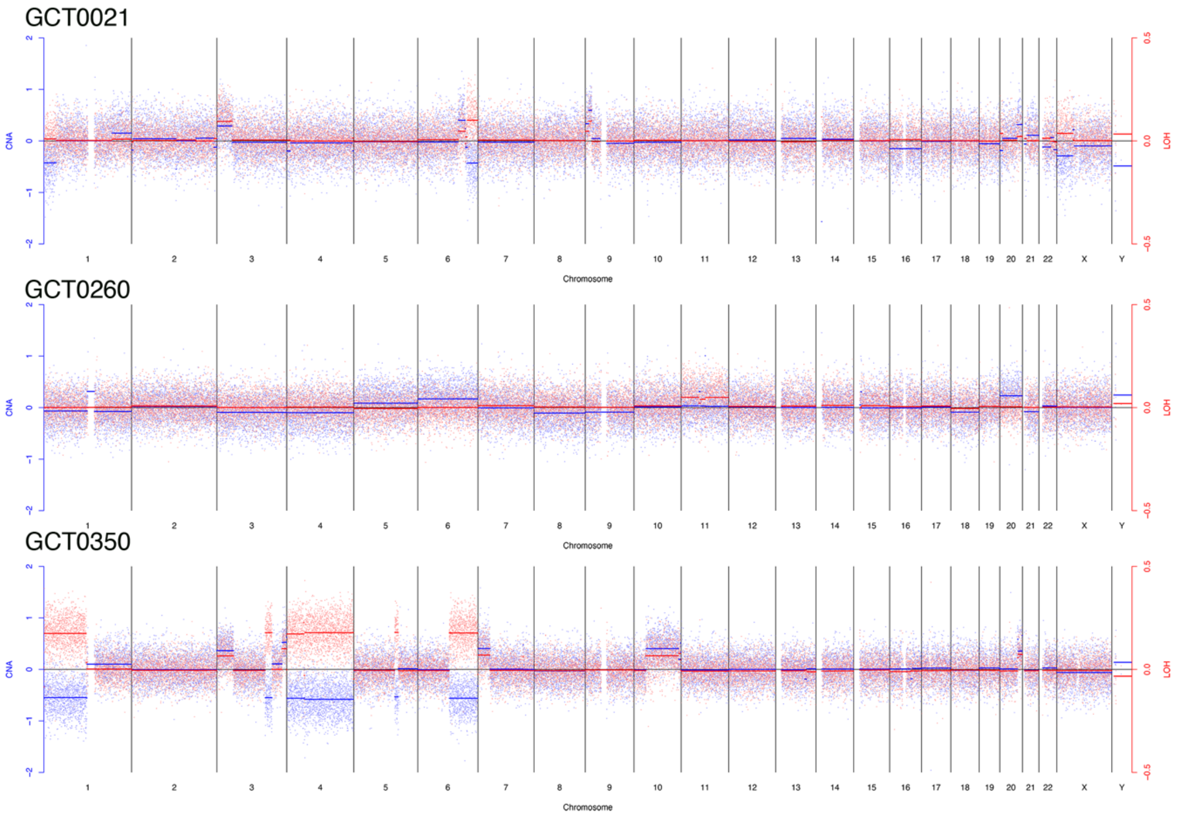

Supplementary Figure 3. Copy-number gains and losses and Loss of Heterozygosity events in yolk sac tumors as determined by whole genome sequencing.

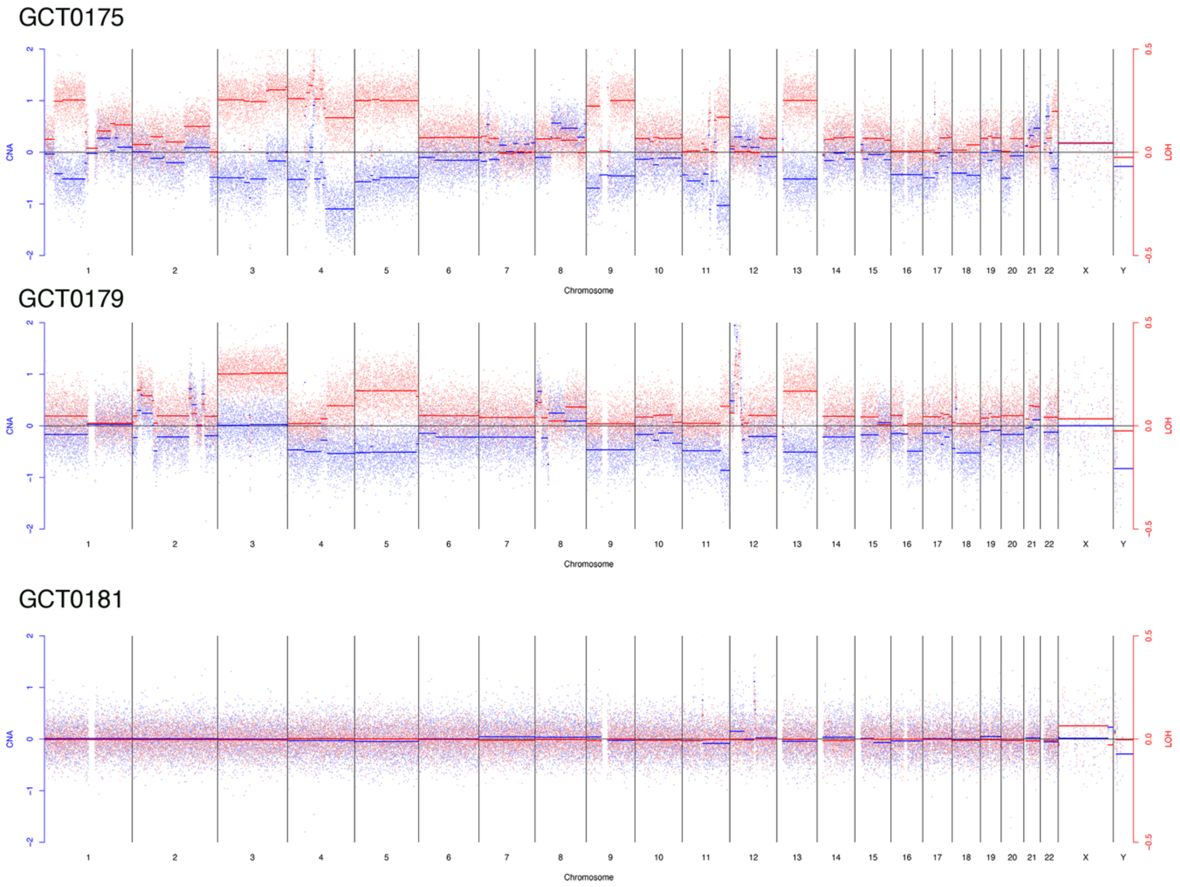

Supplementary Figure 4. Copy-number gains and losses and Loss of Heterozygosity events in germinomas as determined by whole genome sequencing.

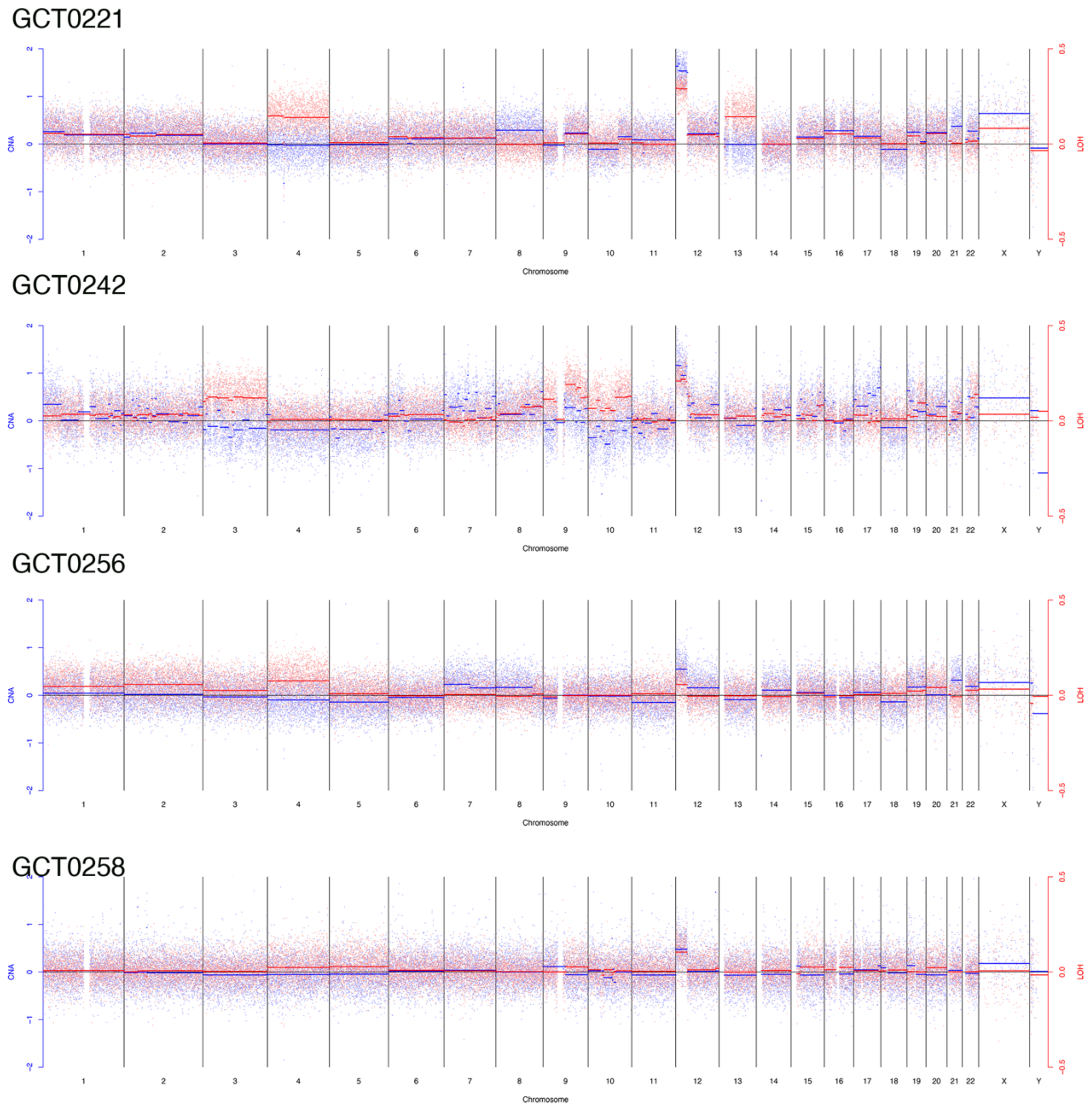

Supplementary Figure 5. Copy-number gains and losses and Loss of Heterozygosity events in embryonal carcinomas as determined by whole genome sequencing.

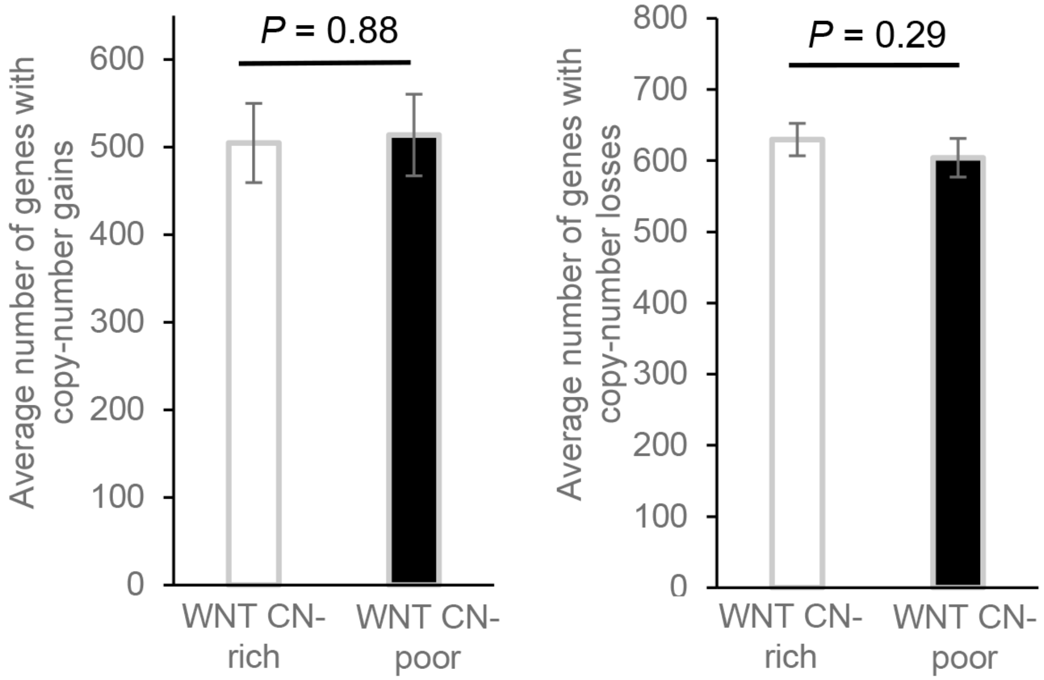

Supplementary Figure 6. Average number of genes with copy-number gains (left) or copy-number losses (right) in TCGA testis tumors stratified into tumors with >5 (WNT CN-rich) and 0-5 (WNT CN-poor) copy-number changes in WNT pathway genes. (WTN CN-rich: n = 85; WNT CN-poor: n= 71 biologically independent tumors). Data are presented as mean values +/- SEM. Two-sided Student's t-test; p=0.88 for genes with copy-number gains; p=0.29 for genes with copy-number losses.

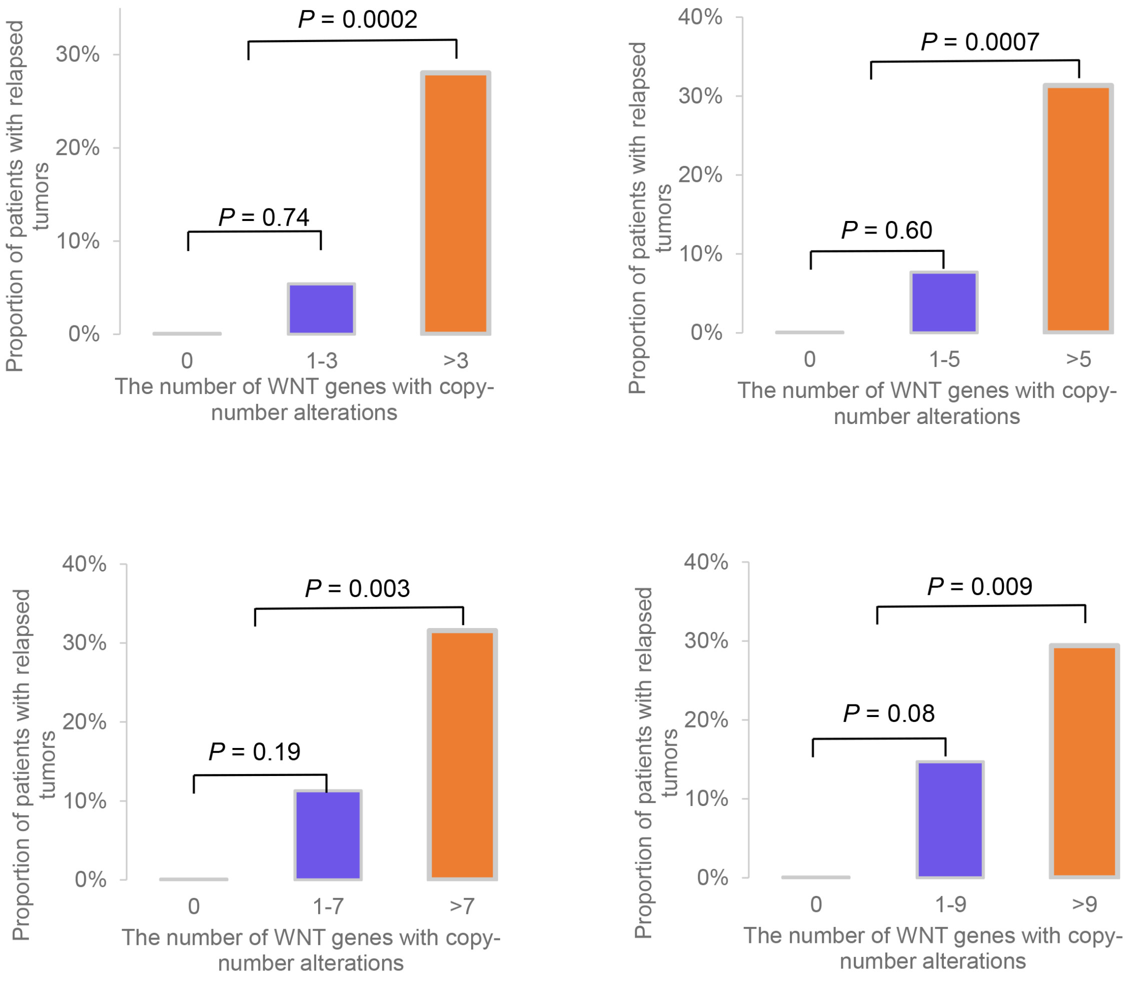

Supplementary Figure 7. Proportion of patients with relapsed germ cell tumors stratified according to the number of WNT pathways genes with WNT gene copy-number alterations (defined as gain of WNT activators or loss of WNT repressors). n = 114 biologically independent tumors. Two-sided Student's t-test; *p*-values as indicated in the figure.

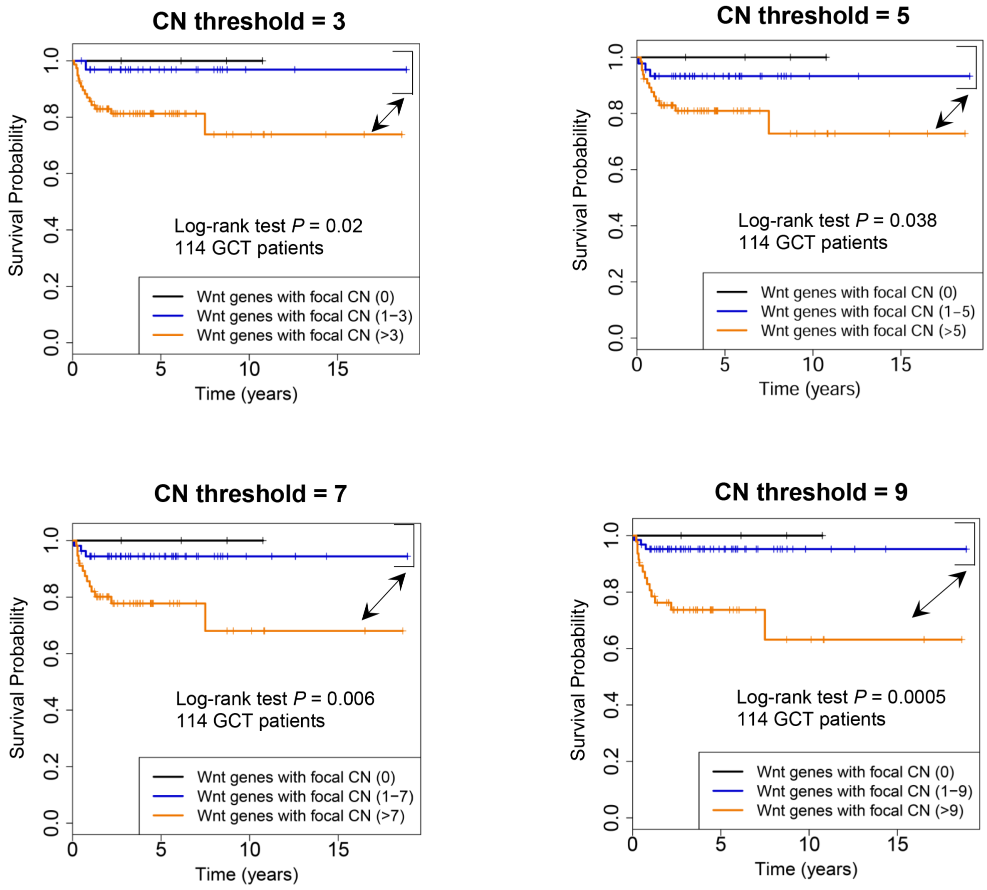

Supplementary Figure 8. Probability of survival of patients with germ cell tumors stratified according to the number of WNT pathways genes with WNT gene copy-number alterations (defined as gain of WNT activators or loss of WNT repressors). n = 114 biologically independent tumors. *p*-values as indicated in the figure.

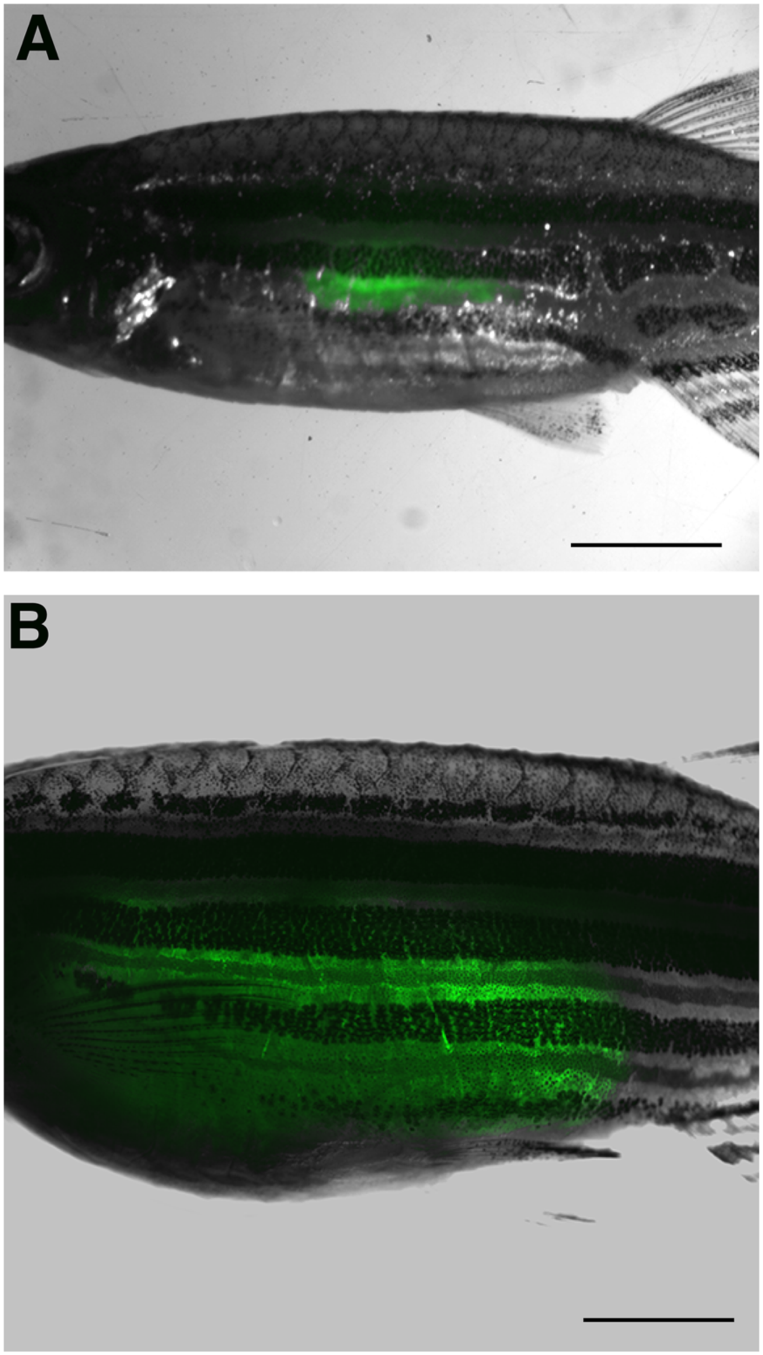

Supplementary Figure 9. Live visualization of germ cells in Tg(*piwil1:eGFP*) zebrafish; lateral view. **A**, wildtype male. GFP fluorescence indicated normal testis. **B**, *bmpr1bb* male with GCT. Expanded GFP corresponds to the area of the tumor. Scale bars: 0.5 cm.

Supplementary Tables

Supplementary Table 1. Germ cell tumor specimens in this study

Patient ID	Type 1/ Type 2	Histology	Gender	Age at diagnosis (y)	WES	Targeted deep sequencing	SNP array	RNA seq	Methylation array	WGS
GCT0001	1	YST	F	2.6		x	x		x	
GCT0002	1	YST	F	2.1	x		x	x	x	
GCT0003	1	YST	M	1.3	x	x	x		x	
GCT0004	1	YST	F	1.3	x	x	x	x		
GCT0005	1	YST	F	16.8		x	x		x	
GCT0006	2	YST	F	1.4			x	x	x	
GCT0007	2	SEM	F	14.7		x	x	x	x	
GCT0008	2	MMGCT	F	16.1			x	x	x	
GCT0009	1	YST	M	1.3		x	x	x	x	
GCT0010	1	YST	F	4.1	x	x	x		x	
GCT0011	1	YST	M	0.9		x	x		x	
GCT0012	1	YST	M	2.0		x	x		x	
GCT0013	1	YST	M	1.4		x	x		x	
GCT0014	1	YST	M	2.8		x	x		x	
GCT0015	1	YST	F	3.0		x	x		x	
GCT0016	1	YST	M	1.0		x	x		x	
GCT0017	1	YST	M	0.7		x	x		x	
GCT0018	1	YST	F	15.1			x	x	x	
GCT0019	1	YST	M	1.3		x	x	x	x	
GCT0020	1	YST	F	11.7		x	x	x	x	
GCT0021	1	YST	F	0.3	x	x	x		x	x
GCT0022	1	YST	M	2.2		x	x	x	x	
GCT0023	1	YST	M	2.6		x	x		x	
GCT0024	1	YST	M	2.1		x	x	x	x	
GCT0025	1	YST	M	2.8		x	x	x	x	
GCT0026	1	YST	unknown	unknown	x		x		x	
GCT0027	1	YST	F	0.6		x	x		x	
GCT0029	1	YST	F	19.6			x	x	x	
GCT0030	1	YST	M	0.3		x	x	x	x	
GCT0031	1	YST	F	12.2			x			
GCT0032	1	YST	M	2.0		x	x	x	x	
GCT0033	1	YST	F	1.4			x		x	
GCT0034	1	YST	M	2.7		x	x	x	x	
GCT0035	1	YST	F	15.2		x	x	x	x	
GCT0036	1	YST	M	3.4		x	x	x	x	
GCT0037	2	YST	M	14.1		x	x	x	x	
GCT0038	1	YST	M	0.8		x	x	x	x	

GCT0039	1	YST	M	0.9	x	x	x	x	x	
GCT0040	2	MMGCT	F	11.4		x	x	x	x	
GCT0041	1	YST	F	11.3	x	x	x	x	x	
GCT0042	2	MMGCT	M	17			x		x	
GCT0045	1	YST	M	0.8			x		x	
GCT0047	2	SEM	F	5				x		
GCT0048	2	MMGCT	M	9			x		x	
GCT0049	2	YST	F	16			x		x	
GCT0050	2	MMGCT	M	16			x		x	
GCT0051	2	EC	M	22			x		x	
GCT0052	2	Teratoma	M	16			x		x	
GCT0053	2	MMGCT	M	18			x		x	
GCT0054	2	SEM	F	14			x		x	
GCT0057	1	YST	M	0			x		x	
GCT0058	2	EC	M	18			x		x	
GCT0059	2	MMGCT	F	19			x		x	
GCT0060	2	MMGCT	M	18			x		x	
GCT0061	2	MMGCT	M	15			x		x	
GCT0062	1	Teratoma	F	0			x		x	
GCT0063	1	YST	?	0.1			x		x	
GCT0064	2	EC	M	19			x		x	
GCT0065	1	Teratoma	?	0			x		x	
GCT0066	1	YST	F	13			x		x	
GCT0067	2	SEM	M	15			x		x	
GCT0068	2	SEM	M	8			x		x	
GCT0069	1	YST	M	1			x		x	
GCT0070	1	Teratoma	F	1			x		x	
GCT0071	2	MMGCT	M	17					x	
GCT0072	2	MMGCT	M	22			x		x	
GCT0073	1	YST	M	0			x		x	
GCT0074	2	MMGCT	M	18			x		x	
GCT0075	2	MMGCT	M	18					x	
GCT0078	2	SEM	F	7					x	
GCT0079	2	SEM	F	16			x		x	
GCT0080	2	MMGCT	M	18			x		x	
GCT0081	1	YST	M	0	x	x	x		x	
GCT0083	2	SEM	F	10			x	x	x	
GCT0084	2	EC	M	16		x	x		x	
GCT0085	2	MMGCT	M	17		x	x		x	
GCT0086	2	SEM	F	11		x	x	x	x	
GCT0087	2	SEM	F	13			x			
GCT0089	2	SEM	F	8			x		x	
GCT0090	2	SEM	F	13		x	x	x	x	
GCT0091	2	SEM	F	14			x		x	
GCT0092	2	SEM	F	7			x			

GCT0093	2	SEM	F	17		x	x	x	x	
GCT0095	2	SEM	M	16			x		x	
GCT0096	2	SEM	M	14			x			
GCT0097	2	SEM	M	14			x		x	
GCT0098	2	SEM	M	14					x	
GCT0099	2	SEM	M	14			x			
GCT0100	2	MMGCT	M	16			x		x	
GCT0101	2	MMGCT	M	16		x	x		x	
GCT0102	2	MMGCT	M	14		x	x		x	
GCT0103	2	MMGCT	F	11			x			
GCT0104	2	MMGCT	M	15			x		x	
GCT0105	1	YST	M	2			x			
GCT0106	2	MMGCT	M	14		x	x		x	
GCT0107	2	MMGCT	F	11		x	x		x	
GCT0108	2	MMGCT	M	15			x		x	
GCT0109	2	MMGCT	M	15		x			x	
GCT0110	1	YST	M	1			x			
GCT0111	1	YST	M	1		x	x			
GCT0112	1	YST	M	1			x			
GCT0113	1	YST	F	8			x		x	
GCT0114	2	YST	F	13			x			
GCT0115	1	YST	M	1		x	x		x	
GCT0116	1	YST	M	3		x	x	x	x	
GCT0117	1	YST	F	1		x	x	x	x	
GCT0118	1	YST	M	1			x		x	
GCT0119	1	YST	M	2		x	x			
GCT0120	1	YST	M	0.9	x	x	x	x	x	
GCT0121	1	YST	M	2			x			
GCT0122	1	YST	M	0		x	x		x	
GCT0123	1	YST	F	0		x	x		x	
GCT0124	2	YST	F	16		x	x	x	x	
GCT0125	1	YST	M	0.5	x	x	x		x	
GCT0126	1	YST	F	16	x	x	x			
GCT0128	2	MMGCT	M	15			x			
GCT0129	2	MMGCT	M	20			x			
GCT0130	2	MMGCT	M	19			x			
GCT0131	2	MMGCT	M	15			x			
GCT0133	2	MMGCT	M	20			x			
GCT0134	2	MMGCT	F	7			x			
GCT0137	2	EC	M	16			x			
GCT0138	1	YST	M	0.5			x			
GCT0142	1	YST	M	1.3			x			
GCT0143	1	YST	F				x			
GCT0144	1	YST	F	12			x			
GCT0145	2	EC	M	17			x			

GCT0146	2	SEM	F	15			x			
GCT0147	2	SEM	M	9			x			
GCT0149	2	SEM	F	5			x			
GCT0152	2	EC	M	20			x			
GCT0153	2	SEM	M	17			x			
GCT0155	2	SEM	F	6			x			
GCT0158	2	SEM	M	15			x			
GCT0159	2	SEM	M	17			x			
GCT0160	2	SEM	M	8			x			
GCT0161	1	YST	M	2			x			
GCT0162	1	YST	M	1.5		x				
GCT0163	1	YST	M	3		x				
GCT0164	1	**YST**	F	0.5		x				
GCT0165	1	**YST**	F	0		x				
GCT0166	1	YST	F	1			x			
GCT0167	1	YST	F	1		x				
GCT0168	1	YST	F	30			x			
GCT0169	1	YST	F	37		x				
GCT0170	1	**YST**	F	0		x				
GCT0174	2	SEM	M	22		x				
GCT0175	2	SEM	M	23	x	x				x
GCT0177	2	SEM	M	21		x				
GCT0179	2	SEM	M	19	x	x				x
GCT0181	2	SEM	M	21	x	x				x
GCT0183	2	SEM	M	23	x	x				
GCT0185	2	SEM	M	23		x				
GCT0186	2	SEM	M	22			x			
GCT0188	2	SEM	M	23			x			
GCT0189	2	SEM	M	22		x				
GCT0190	2	SEM	M	22		x				
GCT0191	2	SEM	M	22		x				
GCT0192	2	SEM	M	20			x			
GCT0193	2	SEM	M	19	x	x				
GCT0196	2	SEM	M	23		x				
GCT0197	2	SEM	M	19		x				
GCT0199	2	SEM	M	23	x	x				
GCT0202	2	SEM	M	21	x	x				
GCT0204	2	SEM	M	23		x				
GCT0205	2	SEM	M	16		x				
GCT0206	2	SEM	M	23			x			
GCT0208	2	SEM	M	18	x	x				
GCT0210	2	SEM	M	22		x				
GCT0211	2	EC	M	16		x				
GCT0214	2	EC	M	22			x			
GCT0216/GCT0217	2	EC	M	23		x				

GCT0219	2	EC	M	22		x				
GCT0220	2	EC	M	19		x				
GCT0221	2	EC	M	18	x	x				x
GCT0223	2	YST	M	17		x				
GCT0225	2	EC	M	23	x	x				
GCT0227	2	MMGCT	M	20	x	x				
GCT0229	2	MMGCT	M	17		x				
GCT0232	2	MMGCT	M	14	x	x				
GCT0234	2	EC	M	23	x	x				
GCT0238	2	EC	M	18	x	x				
GCT0240	2	MMGCT	M	21		x				
GCT0241	2	EC	M	20		x				
GCT0242	2	EC	M	19	x	x				x
GCT0246	2	MMGCT	M	23		x				
GCT0249	2	MMGCT	M	16		x				
GCT0251	2	MMGCT	M	23			x			
GCT0252	2	EC	M	21		x				
GCT0253	2	EC	M	22		x				
GCT0254	2	MMGCT	M	19	x	x				
GCT0256	2	EC	M	18	x	x				x
GCT0258	2	EC	M	20	x	x				x
GCT0260	2	YST	F	21	x	x				
GCT0262	2	EC	M	22		x				
GCT0263	2	MMGCT	M	13		x				
GCT0265	2	YST	M	22			x			
GCT0266	2	EC	M	22			x			
GCT0267	2	EC	M	20		x				
GCT0268	2	EC	M	23	x	x				
GCT0272	2	MMGCT	M	18	x	x				
GCT0274	2	MMGCT	M	19	x	x				
GCT0276	2	MMGCT	M	23			x			
GCT0278	2	MMGCT	M	23			x			
GCT0279	2	MMGCT	M	23	x	x				
GCT0285	2	MMGCT	M	19	x	x				
GCT0288	2	MMGCT	M	23		x				
GCT0289	2	MMGCT	M	21	x	x				
GCT0292	2	MMGCT	M	21			x			
GCT0293	2	MMGCT	M	23	x	x				
GCT0297	2	SEM	M				x			
GCT0298	2	SEM	M				x			
GCT0299	2	SEM	M				x			
GCT0303	2	YST	M				x			
GCT0321	1	YST	M			x				
GCT0322	1	YST	M	2.5	x	x				
GCT0328	2	Teratoma	M	20	x	x				

GCT0330	1	YST	F	1.5	x	x				
GCT0332	1	YST	F	9	x	x				
GCT0338	2	SEM	F	12	x	x				
GCT0340	1	YST	M	1	x	x				
GCT0342	2	SEM	F	11	x	x				
GCT0344	1	Teratoma	M	0	x	x				
GCT0346	1	Teratoma	F	4	x	x				
GCT0348	2	SEM	F	12	x	x				
GCT0350	1	YST	F	2	x	x				x
GCT0352	1	Teratoma	F	14	x	x				
GCT0354	1	Teratoma	F	1		x				
GCT0358	1	YST	F			x				

Abbreviations: WES: whole-exome sequencing of tumor/normal pairs. WGS: whole-genome sequencing. SEM: seminoma. YST: yolk sac tumor. EC: embryonal carcinoma. MMGCT: mixed malignant germ cell tumor. F: female. M: male.

Supplementary Table 2. Whole-exome sequencing statistics

Patient ID	Sample	Sequencing Method	% Reads Mapped	Average depth (X)
GCT0002	tumor	Exome sequencing	96.4%	102
GCT0002	matched normal	Exome sequencing	97.7%	81
GCT0003	tumor	Exome sequencing	95.9%	73
GCT0003	matched normal	Exome sequencing	97.8%	99
GCT0004	tumor	Exome sequencing	97.6%	114
GCT0004	matched normal	Exome sequencing	97.6%	123
GCT0010	tumor	Exome sequencing	97.9%	122
GCT0010	matched normal	Exome sequencing	97.6%	104
GCT0021	tumor	Exome sequencing	97.6%	116
GCT0021	matched normal	Exome sequencing	97.9%	106
GCT0026	tumor	Exome sequencing	92.2%	67
GCT0026	matched normal	Exome sequencing	98.0%	113
GCT0039	tumor	Exome sequencing	96.8%	82
GCT0039	matched normal	Exome sequencing	97.5%	129
GCT0041	tumor	Exome sequencing	97.3%	81
GCT0041	matched normal	Exome sequencing	97.1%	103
GCT0081	tumor	Exome sequencing	98.2%	141
GCT0081	matched normal	Exome sequencing	98.2%	159
GCT0120	tumor	Exome sequencing	98.5%	149
GCT0120	matched normal	Exome sequencing	98.4%	176
GCT0125	tumor	Exome sequencing	98.4%	149
GCT0125	matched normal	Exome sequencing	98.2%	113
GCT0126	tumor	Exome sequencing	97.9%	190
GCT0126	matched normal	Exome sequencing	98.3%	182
GCT0175	tumor	Exome sequencing	97.8%	163
GCT0175	matched normal	Exome sequencing	99.0%	181
GCT0179	tumor	Exome sequencing	97.7%	103
GCT0179	matched normal	Exome sequencing	97.9%	74
GCT0181	tumor	Exome sequencing	96.5%	131
GCT0181	matched normal	Exome sequencing	98.3%	114
GCT0183	tumor	Exome sequencing	98.7%	122
GCT0183	matched normal	Exome sequencing	97.9%	116
GCT0193	tumor	Exome sequencing	97.3%	79
GCT0193	matched normal	Exome sequencing	98.8%	129
GCT0199	tumor	Exome sequencing	94.0%	130
GCT0199	matched normal	Exome sequencing	96.4%	111
GCT0202	tumor	Exome sequencing	97.5%	71
GCT0202	matched normal	Exome sequencing	98.3%	64
GCT0208	tumor	Exome sequencing	98.0%	121
GCT0208	matched normal	Exome sequencing	98.5%	158
GCT0221	tumor	Exome sequencing	98.5%	105

GCT0221	matched normal	Exome sequencing	98.5%	186
GCT0225	tumor	Exome sequencing	98.7%	131
GCT0225	matched normal	Exome sequencing	98.7%	92
GCT0227	tumor	Exome sequencing	98.3%	98
GCT0227	matched normal	Exome sequencing	98.5%	79
GCT0232	tumor	Exome sequencing	97.4%	146
GCT0232	matched normal	Exome sequencing	98.9%	130
GCT0234	tumor	Exome sequencing	97.1%	140
GCT0234	matched normal	Exome sequencing	98.9%	132
GCT0238	tumor	Exome sequencing	98.5%	134
GCT0238	matched normal	Exome sequencing	97.6%	130
GCT0242	tumor	Exome sequencing	98.7%	118
GCT0242	matched normal	Exome sequencing	98.6%	119
GCT0254	tumor	Exome sequencing	92.7%	103
GCT0254	matched normal	Exome sequencing	98.5%	134
GCT0256	tumor	Exome sequencing	97.9%	114
GCT0256	matched normal	Exome sequencing	97.8%	125
GCT0258	tumor	Exome sequencing	98.5%	110
GCT0258	matched normal	Exome sequencing	98.4%	134
GCT0260	tumor	Exome sequencing	98.4%	144
GCT0260	matched normal	Exome sequencing	98.7%	149
GCT0268	tumor	Exome sequencing	96.8%	63
GCT0268	matched normal	Exome sequencing	97.6%	212
GCT0272	tumor	Exome sequencing	95.1%	206
GCT0272	matched normal	Exome sequencing	97.7%	221
GCT0274	tumor	Exome sequencing	92.4%	143
GCT0274	matched normal	Exome sequencing	99.1%	97
GCT0279	tumor	Exome sequencing	98.1%	105
GCT0279	matched normal	Exome sequencing	99.0%	114
GCT0285	tumor	Exome sequencing	97.6%	99
GCT0285	matched normal	Exome sequencing	98.6%	134
GCT0289	tumor	Exome sequencing	96.7%	158
GCT0289	matched normal	Exome sequencing	98.1%	141
GCT0293	tumor	Exome sequencing	97.9%	154
GCT0293	matched normal	Exome sequencing	97.3%	166
GCT0322	tumor	Exome sequencing	60.8%	136
GCT0322	matched normal	Exome sequencing	82.5%	117
GCT0328	tumor	Exome sequencing	68.1%	142
GCT0328	matched normal	Exome sequencing	78.4%	127
GCT0330	tumor	Exome sequencing	54.4%	139
GCT0330	matched normal	Exome sequencing	77.2%	140
GCT0332	tumor	Exome sequencing	99.0%	190
GCT0332	matched normal	Exome sequencing	76.8%	129
GCT0338	tumor	Exome sequencing	70.9%	138
GCT0338	matched normal	Exome sequencing	98.3%	274

GCT0340	tumor	Exome sequencing	99.0%	174
GCT0340	matched normal	Exome sequencing	81.5%	133
GCT0342	tumor	Exome sequencing	55.1%	155
GCT0342	matched normal	Exome sequencing	72.9%	121
GCT0344	tumor	Exome sequencing	64.3%	119
GCT0344	matched normal	Exome sequencing	80.6%	129
GCT0346	tumor	Exome sequencing	77.1%	131
GCT0346	matched normal	Exome sequencing	77.2%	132
GCT0348	tumor	Exome sequencing	60.9%	124
GCT0348	matched normal	Exome sequencing	83.5%	141
GCT0350	tumor	Exome sequencing	81.9%	143
GCT0350	matched normal	Exome sequencing	76.9%	125
GCT0352	tumor	Exome sequencing	72.4%	129
GCT0352	matched normal	Exome sequencing	80.2%	141

Supplementary Table 3. Activators and Repressors of WNT signaling assessed for copy number variation and differential methylation

Activators	Repressors
AKT1	AES
BCL9	APC
CALCOCO1	AXIN2
CTNNB1	BRD7
DAAM1	CHD8
DVL1	CSNK1A1
DVL2	CSNK2A1
DVL3	CTBP1
FZD1	CTNNBIP1
FZD2	CXXC4
FZD3	DKK2
FZD4	DKK3
FZD5	DKK4
FZD7	FAT1
FZD8	FBXW11
FZD9	FBXW2
HNF1A	GPR177
LRP6	GSK3A
LRRFIP2	GSK3B
PYGO1	HDAC1
PYGO2	KREMEN1
RHOU	KREMEN2
ROR1	NKD1
ROR2	PPP2CA
RSPO4	PRKCB
RUVBL1	SFRP2
RUVBL2	TLE3
RYK	ZBTB33
SMAD2	
TCF7L1	
TCF7L2	
WNT1	
WNT11	
WNT2	
WNT2B	
WNT3	
WNT5A	
WNT6	
WNT7A	
WNT7B	

WNT8A	
WNT9A	

Designation of activator vs repressor is according to curation by the R. Nusse lab (http://web.stanford.edu/group/nusselab/cgi-bin/wnt/)

Supplementary Data

Supplementary Data 1. A GCT-relevant list of somatic mutations confirmed by both exome-seq and targeted deep-seq

Supplementary Data 2. Gene fusions predicted from RNASeq
