# Supplementary material for: Integrated genomic analysis reveals aberrations in WNT signaling in germ cell tumors of childhood and adolescence
Source: Nat Commun. 2023 May 6;14:2636. doi: 10.1038/s41467-023-38378-9 (PMC10164134; doi:10.1038/s41467-023-38378-9)
Supplement: Supplementary file 3 — Description of Additional Supplementary Files [file 41467_2023_38378_MOESM3_ESM.pdf]

Description of Additional Supplementary Files

File Name: Supplementary Data 1
Description: A GCT-relevant list of somatic mutations confirmed by both exome-seq and targeted deep-seq

File Name: Supplementary Data 2
Description: Gene fusions predicted from RNASeq
