# Supplementary material for: Integrated genomic analysis reveals aberrations in WNT signaling in germ cell tumors of childhood and adolescence
Source: Nat Commun. 2023 May 6;14:2636. doi: 10.1038/s41467-023-38378-9 (PMC10164134; doi:10.1038/s41467-023-38378-9)
Supplement: Supplementary file 6 — Reporting Summary [file 41467_2023_38378_MOESM6_ESM.pdf]

nature research

Corresponding author(s): Amatruda, James F

Last updated by author(s): 02/09/2023

Reporting Summary

Nature Research wishes to improve the reproducibility of the work that we publish. This form provides structure for consistency and transparency in reporting. For further information on Nature Research policies, see Authors & Referees and the Editorial Policy Checklist.

Statistics

For all statistical analyses, confirm that the following items are present in the figure legend, table legend, main text, or Methods section.

n/a	Confirmed	
☐	☒	The exact sample size (*n*) for each experimental group/condition, given as a discrete number and unit of measurement
☐	☒	A statement on whether measurements were taken from distinct samples or whether the same sample was measured repeatedly
☐	☒	The statistical test(s) used AND whether they are one- or two-sided<br>*Only common tests should be described solely by name; describe more complex techniques in the Methods section.*
☒	☐	A description of all covariates tested
☐	☒	A description of any assumptions or corrections, such as tests of normality and adjustment for multiple comparisons
☐	☒	A full description of the statistical parameters including central tendency (e.g. means) or other basic estimates (e.g. regression coefficient) AND variation (e.g. standard deviation) or associated estimates of uncertainty (e.g. confidence intervals)
☐	☒	For null hypothesis testing, the test statistic (e.g. *F*, *t*, *r*) with confidence intervals, effect sizes, degrees of freedom and *P* value noted<br>*Give P values as exact values whenever suitable.*
☒	☐	For Bayesian analysis, information on the choice of priors and Markov chain Monte Carlo settings
☒	☐	For hierarchical and complex designs, identification of the appropriate level for tests and full reporting of outcomes
☒	☐	Estimates of effect sizes (e.g. Cohen's *d*, Pearson's *r*), indicating how they were calculated

Our web collection on statistics for biologists contains articles on many of the points above.

Software and code

Policy information about availability of computer code

Data collection: No software was used

Data analysis: For whole-exome sequencing data, raw reads was mapped to human reference genome (hg19) using BWA (version bwa-0.7.17). Local realignment and base quality recalibration were performed using default parameters by GATK pipeline (version 3.7). Matched tumor-normal BAM files were used as input for VarScan software (version 2.4.0) to identify somatic single-nucleotide variants (SNVs) and small-scale insertion/deletions (INDELs). For RNA sequencing data, The generated FASTQ files were aligned by Bowtie2 (version 2.3) and TopHat2 (version 2.1.1). Cufflinks (version 2.2.1) was used to assemble and estimate the relative abundances of transcripts at the gene and transcript level. SNP array data was processed by Nexus Copy Number Discovery 7.0 software (BioDiscovery, Inc.). Methylation array data was processed by methylumi package (version 3.7) and IMA package (version 3.1.2). Image analysis was performed with ImageJ (version 1.53m).

For manuscripts utilizing custom algorithms or software that are central to the research but not yet described in published literature, software must be made available to editors/reviewers. We strongly encourage code deposition in a community repository (e.g. GitHub). See the Nature Research guidelines for submitting code & software for further information.

Data

Policy information about availability of data

All manuscripts must include a data availability statement. This statement should provide the following information, where applicable:

- Accession codes, unique identifiers, or web links for publicly available datasets
- A list of figures that have associated raw data
- A description of any restrictions on data availability

Genomic sequencing data of germ cell tumor samples in this study are deposited to dbGaP (https://www.ncbi.nlm.nih.gov/gap/) with Accession Number phs002009.v1.p1. Tumor methylation data are deposited in the Gene Expression Omnibus (GEO) repository under Accession GSE183798. The following databases were used in the analysis: 1000 Genome Project database (https://www.internationalgenome.org); National Heart, Lung, and Blood Institute (NHLBI) Exome Variant

Server (ESP6500; https://evs.gs.washington.edu/EVS/); and the Catalogue of Somatic Mutations in Cancer (COSMIC; https://cancer.sanger.ac.uk/cosmic). Source data are provided with this paper.

Field-specific reporting

Please select the one below that is the best fit for your research. If you are not sure, read the appropriate sections before making your selection.

☒ Life sciences ☐ Behavioural & social sciences ☐ Ecological, evolutionary & environmental sciences

For a reference copy of the document with all sections, see nature.com/documents/nr-reporting-summary-flat.pdf

Life sciences study design

All studies must disclose on these points even when the disclosure is negative.

Sample size	Genomic profiling of tumors was performed to determine the landscape of somatic mutations in pediatric germ cell tumors. No sample size calculation was performed. Malignant germ cell tumor is a rare diagnosis and represents less than 2% of all childhood cancers. The cases profiled represent all cases for which informed consent had been obtained, deidentified clinical annotation was available, and a specimen of suitable sample quality was available in the biospecimen repositories of UT Southwestern Medical Center, Boston Children's Hospital, the Erasmus University Medical Center and the Hospital Sant Joan de Déu, Barcelona, Spain as well as the Children's Oncology Group Biopathology Center, since 2009. This study is - to our knowledge - the largest cohort of pediatric germ cell tumors described to date. For experiments involving animal models, a power calculation was performed using ClinCalc (https://clincalc.com/stats/samplesize.aspx) and sample sizes were chosen to provide 80% to detect a difference in means of 30% with alpha = 0.05.
Data exclusions	No data were excluded during the analysis
Replication	Cell growth experiments were performed using two different small-molecule WNT inhibitors with different mechanisms of action. 4 indepdendent cell lines were used and all cell lines were validated by STR genotyping and confirmed to be free of mycoplasma contamination. Cell growth studies were performed in triplicate. All experiments for cell growth, immunostaining and in vivo drug treatment studies were replicated twice and all replicates were successful.
Randomization	Zebrafish in vivo studies: Animals with tumors were randomly assigned to vehicle control or drug treatment groups. For analysis of human tumor specimens, for some analyses tumors were classified as Type I or Type II according to established criteria as described in the manuscript. Randomization does not apply as there were no within-group comparisons.
Blinding	All measurements including cell line drug exposures, GFP fluorescence images and gH2AX immunostains in drug-treated animals were quantified by personnel blinded to the treatment arm. For bioinformatic analyses, all samples were deidentified at the source and assigned a study number; bioinformatics analysis was performed without reference to associated clinical data except for analyses that compared Type I and Type II tumors.

Reporting for specific materials, systems and methods

We require information from authors about some types of materials, experimental systems and methods used in many studies. Here, indicate whether each material, system or method listed is relevant to your study. If you are not sure if a list item applies to your research, read the appropriate section before selecting a response.

Materials & experimental systems

n/a	Involved in the study	
☒	☐	Antibodies
☐	☒	Eukaryotic cell lines
☒	☐	Palaeontology
☐	☒	Animals and other organisms
☐	☒	Human research participants
☒	☐	Clinical data

Methods

n/a	Involved in the study	
☒	☐	ChIP-seq
☒	☐	Flow cytometry
☒	☐	MRI-based neuroimaging

Eukaryotic cell lines

Policy information about cell lines

Cell line source(s)	Human Germ Cell Tumor Cell line NTERA2 was acquired from the American Type Culture Collection (ATCC). Tcam-2 cells were the gift of Dr. Sohei Kitazawa (Kobe University, Japan). GCT44 and 1411H were provided by Drs. Nick Coleman and Matthew Murray (University of Cambridge, UK). 1411H cells are a patient-derived cell line developed by Voglezang et al. (Vogelzang NJ, Bronson D, Savino D, Vessella RL, Fraley EF. A human embryonal-yolk sac carcinoma model system in athymic mice. Cancer.

1985 Jun 1;55(11):2584-93. doi: 10.1002/1097-0142(19850601)55:11<2584::aid-cncr2820551110>3.0.co;2-b. PMID: 2581684.)

Authentication

All cell lines were validated by STR genotyping.

Mycoplasma contamination

All cell lines tested negative for mycoplasma contamination

Commonly misidentified lines
(See ICLAC register)

No commonly misidentified cell lines were used in this study.

Animals and other organisms

Policy information about studies involving animals; ARRIVE guidelines recommended for reporting animal research

Laboratory animals

Zebrafish (Danio rerio) aged 6-8 months old were used for all experiments. The testicular germ cell tumor phenotype occurs in male animals, therefore males were used for the research.

Wild animals

No wild animals were used in the study

Field-collected samples

No field-collected samples were used in the study

Ethics oversight

All work was performed under protocols approved by the Institutional Animal Care and Use Committee at UT Southwestern Medical Center, an AALAC-accredited institution.

Note that full information on the approval of the study protocol must also be provided in the manuscript.

Human research participants

Policy information about studies involving human research participants

Population characteristics

A total of 229 patients with germ cell tumors (GCTs) were enrolled in this study. Tumor samples and clinical information used in this study were obtained under informed consent and approval by the Institutional Review Board of the participating facility. Samples were assembled from collections at the University of Texas Southwestern Medical Center, Dallas, TX USA; Children's Oncology Group; Boston Children’s Hospital, Boston, MA USA; the Erasmus University Medical Center, Rotterdam, Netherlands; and the Hospital Sant Joan de Déu, Barcelona, Spain. All samples were de-identified at the source.

Recruitment

No recruitment was done for this study.

Ethics oversight

All patients gave informed consent for the use to tissues for research. All samples were collected under IRB-approved protocols at the governing Ethics Oversight or Institutional Review Board of the participating facilities at the University of Texas Southwestern Medical Center, Dallas, TX USA; Children's Oncology Group; Boston Children’s Hospital, Boston, MA USA; the Erasmus University Medical Center, Rotterdam, Netherlands; and the Hospital Sant Joan de Déu, Barcelona, Spain. All samples were de-identified at the source.

Note that full information on the approval of the study protocol must also be provided in the manuscript.
